# Exploring the Indoor Plant–People Relationship Through Qualitative Responses

**DOI:** 10.1002/pei3.70025

**Published:** 2024-12-29

**Authors:** B. Le Busque, C. Litchfield, C. L. Shaw

**Affiliations:** ^1^ Stem University of South Australia Adelaide South Australia Australia; ^2^ Conservation Psychology and Applied Animal Behaviour Research Group, Justice & Society University of South Australia Adelaide South Australia Australia; ^3^ Adelaide Business School University of Adelaide Adelaide South Australia Australia

**Keywords:** biophilia, connection to nature, indoor plants, plant–people relationship, qualitative segmentation

## Abstract

Humans have a long‐standing relationship with the natural world, particularly in how they engage with plants—referred to as people–plant relationships. While plants naturally live outdoors, people have been including them inside built environments for centuries. Although the benefits of indoor plants are well documented in research, there is limited exploration of individuals' subjective relationships with their indoor plants. To address this gap, we examined the perceived benefits of owning indoor plants and how people describe their relationships with them through open‐ended qualitative survey items. Data were collected from 115 indoor plant owners in Australia, and the qualitative responses were analyzed using a combination of thematic analysis and qualitative segmentation. On average, participants owned 15 indoor plants and in total participants identified 11 benefits. The most common being decorative and aesthetic value, improved air quality, and calming effects. Participants fell into one of four types of relationships with their indoor plants: highly connected, engaged, limited engagement, and no relationship. This qualitative segmentation approach allowed us to achieve a key goal of exploratory qualitative research—providing new insights to inform future quantitative studies. Given that we found that not all indoor plant–people relationships are equal, and that people have varying levels of connection to their plants, future research should explore these relationship types using quantitative methods.

## Graphical abstract

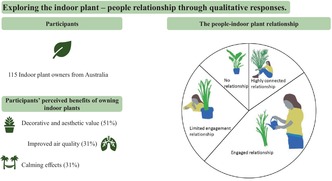

## 1 Introduction

Humans have a long‐standing intimate and intricate relationship with the natural world (Soga and Evans 2024), a connection neatly captured by the Biophilia Hypothesis first proposed by Edward Wilson (Wilson and Kellert 1993). One aspect of this relationship is with plants, forming what is referred to as people–plant interactions (Ikeya and Balée 2023). While plants naturally exist outside of human‐made environments, people have been bringing plants inside for centuries (Bringslimark, Hartig, and Patil 2009). These indoor plants have been valued by the design industry for their aesthetical qualities (Khan and Kamal 2023), but their benefits go beyond aesthetic value.

The diverse benefits of indoor plants are well‐documented in numerous peer‐reviewed studies. As providing a detailed literature review is beyond the scope of this paper, we refer to systematic reviews and key experiments for a summary. A review of experimental literature identified psychological benefits of indoor plants, including stress reduction and increased pain tolerance (Bringslimark, Hartig, and Patil 2009). Ten years later, an updated review synthesized 50 studies and found that the most significant effects of indoor plants were an increase in positive emotions and a reduction in negative feelings, followed by a decrease in physical discomfort (Han and Ruan 2019). A more recent scoping review of six studies concluded that indoor plants generally lead to reduced stress, fewer depressive symptoms, and less negative emotion (Zhao et al. 2023). These benefits have specifically been observed in various settings including office workspaces (Raanaas et al. 2011), hospital rooms (Park and Mattson 2009), and during the COVID‐19 pandemic (Pérez‐Urrestarazu et al. 2021). Importantly for validity, both subjective and objective measures have been used to document these effects. For example, Qin et al. (2014) employed a biological signal measurement device, PowerLab, which recorded parameters such as electroencephalogram (EEG), electrocardiogram (ECG), oxyhemoglobin saturation, fingertip blood flow, skin resistance, and respiration rate to assess changes in participants exposed to various plant conditions in an office. Results showed significant differences in these measurements, with indicators of human comfort observed when participants were exposed to plants. Similarly, Ma et al. (2024) found that objective physiological measures indicated that university students felt less drowsy and more relaxed when exposed to a small “green wall” in an office setting. Research has also explored the mechanisms by which plants contribute to these documented benefits and a review identified four proposed mechanisms: photosynthesis, transpiration, psychological effects, and air purification (Deng and Deng 2018).

While the benefits of indoor plants are well‐documented, less attention has been given to understanding people's awareness of these benefits and how they describe their personal relationships with indoor plants, especially via qualitative data. A study by Berger et al. (2022) investigated how the appearance of indoor plants impacts people's perceptions of subjective wellbeing using a web‐based photo questionnaire. The survey included an open‐ended item where participants (*N* = 203) could provide additional information about their plant preferences. Thematic analysis of these responses revealed that participants commonly described indoor plants using words such as “welcoming,” “joyful,” “happy,” “relaxing,” and “calm,” reflecting their perceived benefits. Similarly, van den Bogerd et al. (2021) studied the presence of indoor plants in study rooms and their effect on mood and cognitive performance. The survey included an open‐ended item where 93 students provided reasons for their preference to study in rooms with plants. The most frequent responses were related to higher perceived environmental quality (*n* = 18), a more pleasant atmosphere (*n* = 18), and a sense of relaxation (*n* = 18). While both Berger et al. (2022) and van den Bogerd et al. (2021) collected qualitative insights through optional open‐ended survey questions, Raanaas et al. (2011) conducted in‐depth individual and group interviews with residents at a rehabilitation center. Respondents reported that thriving indoor plants were pleasant to look at and that these natural elements symbolized health and life. These three studies either included only a single qualitative survey item or focused on a specific demographic within a rehabilitation center. Therefore, there is a need to explore the subjective relationship between people and indoor plants in a general population sample.

Given that exploring human–nature relationships is an emerging field of research (Fukano and Soga 2024), further understanding the relationship between people and plants can help unlock the potential for nature to improve human health and wellbeing (Niazi et al. 2023). This current research, therefore, aims to investigate how people perceive the benefits of owning indoor plants and how they describe their relationship with them, using open‐ended (qualitative) survey items.

## 2 Methods

### 2.1 Sample

The study population comprised a sample of 115 adults (> 18 years), who lived in Australia and were recruited using a snowball sample through social media posts (Instagram, Facebook, and X, formally Twitter) and poster advertisements at the University of South Australia. Data were collected in 2020, and participation in the study was voluntary. Participants were encouraged to take part with the incentive of a chance to win one of two gift cards through a randomized draw. To be included in the study, participants were required to report having indoor plants during the data collection period. The final sample consisted of 68.7% female, 30.5% male, and 0.8% non‐binary participants, with ages ranging from 18 to 69 (mean = 35).

### 2.2 Data Collection

The short survey (see Supporting Information for full survey) included items to collect gender and age demographic data. Data were then collected regarding indoor plant demographics specifically with participants answering how many indoor plants they own (on an open‐ended numerical survey item), which rooms in the house their indoor plants are in (checkbox), and what species of indoor plants they own (open‐ended text item). Participants then answered two open‐ended items, “*What benefits do you experience from having indoor plants inside your house?”* and *“Describe your relationship with your indoor plants.”*

### 2.3 Data Analysis

Plant ownership data were exported and analyzed in Excel. The data on the number of plants owned were sorted in chronological order to determine the range and mean average. One participant reported owning over 500 plants, which represented a large deviation from the mean. However, given the wide range of data and the fact that most of the data were analyzed qualitatively, this entry was not considered an outlier for removal. The plant varieties were sorted and collated to calculate the percentage of participants who owned each variety. Additionally, percentages were calculated to determine which rooms indoor plants were most frequently kept in.

Berger et al. (2022) employed a thematic analysis approach to analyze data obtained from an open‐ended item. The data were collated and organized into themes and subthemes using an inductive thematic analysis approach, meaning that themes emerged directly from the data rather than being pre‐determined (Clarke and Braun 2017). This method was applied to analyze responses to the first open‐ended question: “What benefits do you experience from having indoor plants inside your house?”

For the second open‐ended question, “Describe your relationship with your indoor plants,” the data were again collated and organized (Berger et al. 2022). However, a qualitative segmentation approach was subsequently applied. Rather than simply identifying themes, this approach aimed to group or cluster responses in a way that allowed categorization of participants into distinct segments (Bond and Morris 2003), a method commonly used in the marketing sector (Tynan and Drayton 1987). In this case, the segments were based on the type of relationship participants described with their indoor plants, with each participant being categorized into only one segment. Similar to the inductive thematic analysis approach (Clarke and Braun 2017), the segments were determined through manual analysis of the data rather than using pre‐conceived categories or supervised models.

## 3 Results

### 3.1 Indoor Plant Ownership

There was a large range in the number of indoor plants that participants reported owning, from one plant through to 500+ plants, with a mean average of 15 plants reported. A total of 51 different varieties of indoor plants were owned (see Supporting Information for full list), with the most common varieties being succulents (owned by 32% of participants), Devil's Ivy/Golden Pothos ( *Epipremnum aureum* ) owned by 24% of participants, and monstera ( *Monstera deliciosa* , owned by 20% of participants). Most commonly, participants kept their indoor plants in the living room (75%), followed by the kitchen (54%), and bedroom (50%).

### 3.2 Perceived Benefits of Indoor Plants

**FIGURE 1:**
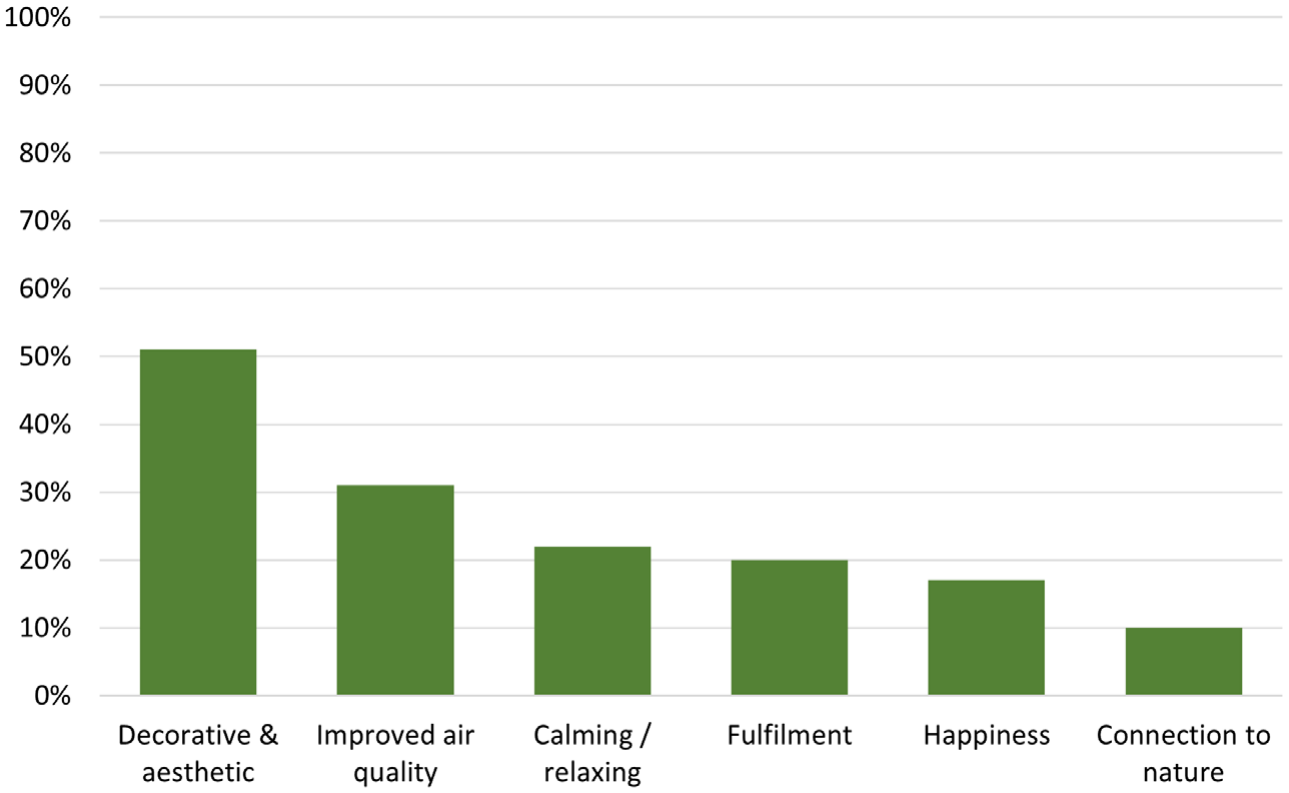
The benefits of having indoor plants that were reported by 10% or more of the participant sample.

In total, participants reported eleven different benefits of having indoor plants, with the benefits reported by 10% or more of the sample being shown in Figure 1. Half of the sample (51%) described the decorative and/or aesthetic benefits of indoor plants including that the “*plants are nice to look at,”* “*soften rooms,”* “*add colour to rooms,”* and “*make the spaces look nice.”*. Benefits to air quality were discussed by 31% of the sample including that their indoor plants “*purify the air*,” “*produce oxygen*,” and “*make the air feel better*.” Just under a quarter of the sample (22%) described how their indoor plants were calming, “*reduced stress*,” and “*relaxed their mind*.” The remaining five benefits described by fewer than 10% of the sample were *plants help them set habits*, *physical health benefits*, *provide distraction*, *relieve fatigue*, and *pleasant smell*.

### 3.3 People's Relationships With Their Indoor Plants

The participants in this sample fell into one of four types of relationships with their indoor plants that we have labeled as highly connected relationship, engaged relationship, limited engagement relationship, and no relationship (Table 1).

**TABLE 1:** Descriptions of examples of the four types of indoor plant–people relationships found in the participant sample.

Type of person‐indoor plant relationship	Definition of relationship	Examples
Highly connected relationship	There is a high connection between the person and their indoor plants, including a love of their indoor plants or even considering these plants to be members of the family.	“They are like my children” [28‐year‐old male participant]
		“I often water them and take care of them as family members” [26‐year‐old female participant]
		“Well I cried over my plants leaf getting broken off today, so you could say I'm pretty attached to her.” [21‐year‐old female participant]
Engaged relationship	The person is emotionally attached to their indoor plants and spends time attending to and taking care of their plants.	“Watering them and watching them grow is exciting, I feel proud to keep them alive so long” [22‐year‐old female participant]
		“I feel satisfied whenever I feed them and then I get seriously nervous when I notice any form of withering” [31‐year‐old female participant]
		“I get sad when one dies or is looking droopy, I feel happy when they look alive and freshly watered” [22‐year‐old female participant]
Limited engagement relationship	The person enjoys having indoor plants, but without an emotional attachment to these plants, and with minimal amount of time spent caring for them.	“Don't have a green thumb so happy when they live” [58‐year‐old female participant]
		“Spend minimal time with the plants” [32‐year‐old female participant]
		“Feel like indoor plants are fine but through our large windows we can see our outdoor plants and that's more important to us” [45‐year‐old female participant]
No relationship	The person has no relationship with their indoor plants	“No relationship. Hardly watered it as it's a succulent.” [21‐year‐old male participant]
		“No relationship—they are all gifts rather than something I've gone out to buy” [21‐year‐old male participant]
		“No relationship with my plants.” [33‐year‐old male participant]

**FIGURE 2:**
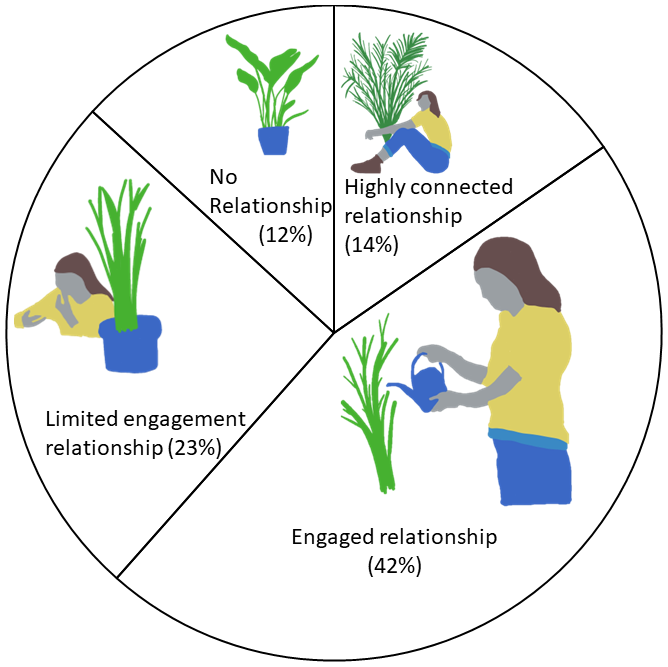
Percentage of the sample that fell into each of the four indoor plant–people relationships.

Most commonly, participants had an engaged relationship with their plants (42%), while approximately a quarter (23%) of participants have a limited engagement relationship with their indoor plants. Fourteen percent of the sample have a highly connected relationship with their indoor plants, and 12% of the sample report having no relationship with their indoor plants (Figure 2).

## 4 Discussion

This study utilized open‐ended qualitative items to explore the relationship between indoor plant owners and their plants. As understanding human–nature relationships is a developing area of research (Fukano and Soga 2024), preliminary studies such as ours that explore qualitative data provide new insights and important avenues for future research.

Our sample reported a total of eleven different benefits of having indoor plants in their homes. The most mentioned benefit was the aesthetic value of plants, which aligns with findings from previous research (Berger et al. 2022; van den Bogerd et al., 2021). Berger et al. (2022) also identified maintenance as a common benefit described by their participants, which corresponds with the fulfillment theme in this study. Similarly, van den Bogerd et al. (2021) highlighted that their participants reported the benefits of a pleasant atmosphere and a sense of relaxation, which align with the improved air quality and calming/relaxing themes identified in this study, respectively. The three most frequently cited benefits in this study, decorative/aesthetic value, improved air quality, and calming effects (which were also found by Berger et al. 2022; van den Bogerd et al., 2021) are all also supported by empirical research. Specifically, studies have found that indoor plants enhance the aesthetic appeal of spaces (e.g., Khan and Kamal 2023), improve air quality (e.g., Deng and Deng 2018), and reduce stress by promoting relaxation (Zhao et al. 2023). This provides evidence that, even when unprompted (e.g., using open‐ended items), participants often report benefits that are empirically supported in the literature. The fourth most frequently mentioned benefit was that indoor plants provide a sense of fulfillment or purpose. While there is limited empirical exploration of this benefit, a qualitative study on garden therapy found that some participants experienced increased self‐esteem and satisfaction from observing the growth of the plants they tended (Adevi and Mårtensson 2013). Further quantitative studies are needed to explore the relationship between indoor plants, fulfillment, and self‐esteem. One benefit, pain tolerance, has been identified in previous research (Bringslimark, Hartig, and Patil 2009) but was not mentioned by any participants in this study or in the studies by Berger et al. (2022) and van den Bogerd et al. (2021). This suggests it would be valuable to further investigate people's perceptions and knowledge regarding the potential of indoor plants to increase pain tolerance, as it seems not to be a commonly recognized benefit.

Using a qualitative segmentation approach to analyzing how the participants described their relationship with indoor plants allowed us to identify four distinct indoor plant–participant relationships. These range from a “highly connected” relationship where people referred to their plants as part of the family and described a deep love for their plants through to “no relationship,” where people described having no relationship with the plants in their house. While a little over 10% of the sample fell into each of these ends of the relationship, most participants described either an engaged relationship or a limited engagement relationship. The qualitative segmentation approach is typically used in marketing, as it allows companies to think of their customers as belonging to different segments and therefore marketing can target these different segments, rather than try to serve all customers in the market the same way (Dibb, Stern, and Wensley 2002). While segmenting the indoor plant–people relationship could allow marketing to serve these segments, it was not the purpose of this study. The segmentation approach was used to achieve one of the purposes of exploratory qualitative research – to provide new insights and prelude quantitative research (Glaser and Strauss 1966).

Therefore, these preliminary results should serve as a foundation for subsequent studies. An important next step is to determine whether the same segment categories identified in this study can be applied to a larger, international sample, as this study was limited to Australians recruited through convenience sampling. These segments should then be used to develop and validate a quantitative scale that measures the indoor plant–person relationship and categorizes participants into one of the identified relationship types. This would allow for further exploration of interesting questions in the human–nature relationship context, such as: Do individuals with highly connected relationships experience greater benefits from indoor plants? Do those with stronger connections to their plants engage in more pro‐environmental behaviors? Are there demographic differences across the different types of relationships?

It is also important to acknowledge that the data for this study were collected in 2020, a year when many people were experiencing the impacts of COVID‐19. A study found that having indoor plants during the COVID‐19 confinement was associated with positive well‐being (Pérez‐Urrestarazu et al. 2021), suggesting that the context of the pandemic may have influenced participants' relationships with indoor plants or their perceptions of the benefits these plants offer. This underscores the importance of replicating this approach in a broader sample post‐COVID‐19 to determine whether there are any differences in the results.

## 5 Conclusion

Continued research into the human–nature relationship is essential in unlocking the full potential of nature to enhance human health and well‐being (Niazi et al. 2023), while also exploring how humans can contribute to the health and well‐being of nature. Through the analysis of qualitative data, this study provides preliminary evidence that individuals are aware of many of the benefits of indoor plants and that not all indoor plant–person relationships are the same. People exhibit varying levels of connection and engagement with their plants, highlighting the diversity in these relationships. This research serves as a foundation for subsequent studies to replicate, validate, and expand upon these findings.

## Conflicts of Interest

The authors declare no conflicts of interest.

## Supporting information


Data S1.

Data S2.

Data S3.

## Data Availability

The data that supports the findings of this study are available in the Supporting Information of this article.
