# Supplementary material for: Exploring the Indoor Plant–People Relationship Through Qualitative Responses
Source: Plant Environ Interact. 2024 Dec 29;5(6):e70025. doi: 10.1002/pei3.70025 (PMC11683047; doi:10.1002/pei3.70025)
Supplement: Supplementary file 2 — Data S2. [file PEI3-5-e70025-s002.docx]

| **Variety of plant** | **Scientific name (if applicable)** | **N** | **%** |
| --- | --- | --- | --- |
| Succulent |  | 37 | 32% |
| Devil Ivy / Golden Pathos | Epipremnum aureum | 28 | 24% |
| Monstera | Monstera deliciosa | 23 | 20% |
| Peace Lily | Spathiphyllum wallisii | 17 | 15% |
| Fiddle-leaf fig | Ficus lyrata | 14 | 12% |
| Rubber fig | Ficus elastica | 13 | 11% |
| A mother in laws toungue | Dracaena trifasciata | 10 | 9% |
| Chinese money plant | Pilea peperomioides | 8 | 7% |
| Fern |  | 8 | 7% |
| Snake plant | Dracaena trifasciata | 6 | 5% |
| Hoya | Hoya | 6 | 5% |
| Palm | Arecaceae | 6 | 5% |
| Spider plant | Chlorophytum comosum | 6 | 5% |
| Bamboo | Bambusa | 5 | 4% |
| Peacock plants | Calathea | 5 | 4% |
| Orchid | Orchidaceae | 4 | 3% |
| Zanzibar gem | Zamioculcas | 4 | 3% |
| Aloe Vera | Aloe vera | 3 | 3% |
| Areca Palm | Dypsis lutescens | 3 | 3% |
| Chinese evergreen | Aglaonema | 3 | 3% |
| Parlour Palm | Chamaedorea elegans | 3 | 3% |
| Polka dot Begonia | Begonia | 2 | 2% |
| Birds of paradise | Strelitzia juncea | 2 | 2% |
| Bromeliad | Bromeliaceae | 2 | 2% |
| Cyclamen | Cyclamen | 2 | 2% |
| Marimo | Aegagropila linnaei | 2 | 2% |
| Pearl of chains | Curio rowleyanus | 2 | 2% |
| Agava / Agave |  | 1 | 1% |
| Australian Tree Fern | Cyathea cooperi | 1 | 1% |
| Bonsai |  | 1 | 1% |
| Brandy plant | Philodendron Brandtianum | 1 | 1% |
| Canna | Canna | 1 | 1% |
| Cliff Orchid | Sarcochilus hartmannii | 1 | 1% |
| Crotons | Codiaeum | 1 | 1% |
| English Ivy | Hedera helix | 1 | 1% |
| Herb |  | 1 | 1% |
| Jewell Orchid | Ludisia | 1 | 1% |
| Lady ferns | Athyrium filix-femina | 1 | 1% |
| Monterey cypress | Hesperocyparis macrocarpa | 1 | 1% |
| Mint | Mentha | 1 | 1% |
| Money Tree | Pachira aquatic | 1 | 1% |
| Flaming Katy | Kalanchoe blossfeldiana | 1 | 1% |
| Prickly pear | Opuntia | 1 | 1% |
| Red anthurium | Anthurium andraeanum | 1 | 1% |
| Rosemary | Salvia rosmarinus | 1 | 1% |
| Dolphin Necklace | senecio peregrinu | 1 | 1% |
| Air plant | Tillandsias | 1 | 1% |
| Tulip | Tulipa | 1 | 1% |
| Umbrella plant | Heptapleurum actinophyllum | 1 | 1% |
| Weeping fig | Ficus benjamina | 1 | 1% |
| Yucca | Asparagaceae | 1 | 1% |
