# Supplementary material for: Exploring the Indoor Plant–People Relationship Through Qualitative Responses
Source: Plant Environ Interact. 2024 Dec 29;5(6):e70025. doi: 10.1002/pei3.70025 (PMC11683047; doi:10.1002/pei3.70025)
Supplement: Supplementary file 3 — Data S3. [file PEI3-5-e70025-s003.docx]

Survey items:

1. Are you 18 years old or older? Yes/ No
2. Please specify your gender: [open ended]
3. Please specify your age: [open ended]
4. Do you have an indoor plant in your home Yes/No
5. If yes, how many indoor plants did you have in your home? [open ended]
6. What species of indoor plants did you have (if known)? [open ended]
7. Did you have indoor plants in your (please specify all that apply):
   - *a. Kitchen*
   - *b. Living room*
   - *c. Lounge room*
   - *d. Bedroom*
   - *e. Bathroom*
   - *f. Home office*
   - *g. Dining room*
   - *h. Other (please specify)*
8. What benefits did you experience from having indoor plants inside your house? [open ended]
9. Describe your relationship with your indoor plants [open ended]
